# Circulating Brain-Derived Neurotrophic Factor (BDNF) and Multimodal Opioid-Based Analgesia in Chronic Pain: Plasma BDNF as an Indicator of Pain Intensity and Neuropathic Pain

**DOI:** 10.3390/biomedicines14061313

**Published:** 2026-06-10

**Authors:** Urszula Kosciuczuk, Piotr Jakubow, Damian Misiuk

**Affiliations:** 1 Department of Anaesthesiology and Intensive Therapy, Medical University of Bialystok, 15-089 Białystok, Poland; 2 Department of Anaesthesiology and Intensive Therapy of Children, Medical University of Bialystok, 15-089 Białystok, Poland

**Keywords:** analgesia, multimodal therapy, pain

## Abstract

**Background:** Brain-derived neurotrophic factor (BDNF) is crucial in the nociception and mechanisms underlying chronic and neuropathic pain. The evaluation of circulating BDNF in patients with multimodal analgesia has not been reported previously. We hypothesized that opioid-based multi-analgesia induces changes in BDNF values and that BDNF correlates with pain intensity in neuropathic pain. **Methods**: Adult patients who met low back pain (LBP) criteria and received multimodal opioid-based therapy were included. The control group included patients with LBP who did not receive any pharmacotherapy. Plasma measurements obtained with the ELISA test were analyzed. The study was registered at Clinical Trials.gov (NCT 04227223). **Results**: Patients with multimodal opioid-based analgesia had significantly higher BDNF values compared to the monotherapy: 3.6 ng/mL vs. 2.7 ng/mL, *p* = 0.01. No statistical differences were observed compared to the non-pharmacologically treated group: 3.6 ng/mL vs. 5.0 ng/mL, *p* = 0.75. The median BDNF values were lowest in the mild-pain group, and significant differences were observed between the severe and moderate-pain groups (*p* = 0.006) and the mild-pain group (*p* = 0.0001). BDNF was significantly higher in the neuropathic-pain group compared to the group of patients without neuropathic pain (*p* = 0.0005). A significant correlation was demonstrated between the BDNF and numerical rating pain score (NRS) in the neuropathic-pain component (rho = 0.6, *p* = 0.001). **Conclusions**: Multimodal opioid-based analgesia decreases plasma BDNF concentrations less than opioid monotherapy, which offers an opportunity to limit opioid-induced adverse effects. BDNF influences pain intensity and predicts neuropathic pain in multimodal opioid-based analgesia.

## 1. Introduction

Since its discovery in 1982, brain-derived neurotrophic factor (BDNF) has been extensively studied for its crucial involvement in nociception and pathological pain mechanisms such as chronic and neuropathic pain. In addition to its well-established functions in peripheral and central pain transmission, BDNF has emerged as a key player in brain neuroplasticity, memory, and cognitive processes [1,2,3,4].

BDNF belongs to a group of neurotrophins and mainly acts through the TrkB–neurotrophic tyrosine kinase family, with a minimal affinity for p75 NRT (p75 neurotrophin receptor), ultimately resulting in postsynaptic depolarization mediated by cAMP and Ca influx. Nociception processes resulting from BDNF/TrkB receptor interactions also coexist with glutamatergic and GABAergic modulation, regulated by the potassium-chloride exporter (KCC2), as well as interactions with endocannabinoid and opioid transmission [4,5,6,7].

BDNF is expressed in all neurons of the nociceptive pathway (sensory neurons in the dorsal root ganglion, spinal dorsal horn, thalamus, and sensory cortex) and in limbic system structures, which regulate affective behaviors in pain perception. At the same time, the neurotrophic effects of BDNF—regulation of differentiation, apoptosis, neurogenesis, and synaptogenesis—are of paramount importance. Experimental induction of traumatic, inflammatory, and pathological pain transmission mechanisms, such as neuropathic pain, have been shown to increase BDNF concentrations directly in neural tissues. BDNF crosses the blood–brain barrier, is detectable in blood, and is significantly correlated with neuronal concentration [8,9,10].

The importance of circulating BDNF in vivo in neurodegenerative and psychiatric disorders is well established, but little is still known about the relationship between analgesic therapy for chronic pain and BDNF concentration [11,12,13,14,15]. Opioid drugs are the most controversial when assessing various analgesic models for chronic pain. Experimental studies have shown that mi opioid receptor agonists regulate BDNF expression and nociception through T-cell immunomodulation and pro-inflammatory cytokine secretion. The first publications on the immunomodulatory effects of opioids on T lymphocytes, neutrophils, and macrophages were described in 1979. Furthermore, T lymphocytes and neuronal cells have a close humoral relationship. Pro-inflammatory cytokines (interleukines- IL-4, IL-5) secreted by T lymphocytes stimulate neuronal VIP (vasoactive intestinal peptide) secretion, increasing the pro-inflammatory activity of CD4+ T lymphocytes through feedback. T lymphocytes stimulate BDNF production through IL-4-mediated effects on neurons. At the same time, T lymphocytes are an important link in opioid receptor interactions, as they have been reported to express all opioid receptors [16,17,18]. Meanwhile, long-term exposure to opioids has been associated with increased neuronal degeneration and local reductions in BDNF expression, accompanied by morphological changes in brain tissue. Another interesting issue is the use of BDNF concentration as a marker of neuropathic pain. Additionally, selectively inhibiting BDNF’s pro-nociceptive effects in pain therapy represents a future research topic [19,20,21,22].

Chronic low back pain is the most frequent medical situation requiring the use of various analgesic models, including opioid-based analgesia. The severity of the social and medical consequences of escalating chronic pain therapy induced by pain intensity progression is crucial.

The present study is a secondary analysis of data from a previously reported single-center, cross-sectional observational study at the Pain Clinic, Bialystok, Poland, which evaluated the effect of opioid monotherapy on BDNF concentration. However, to the best of our knowledge, an evaluation of plasma BDNF levels in patients with multimodal analgesia has not been reported previously. Therefore, this study was designed to investigate BDNF values in patients receiving multimodal opioid-based analgesia. We hypothesized that opioid-based multi-analgesia would induce changes in BDNF values, and that BDNF would be correlated with pain intensity in neuropathic pain. Thus, BDNF measurements would be beneficial to understand the efficacy of opioid-based multi-analgesia in chronic and neuropathic pain. The results of this study are expected to provide insights into the potential effectiveness or side effects of opioid-based analgesia.

## 2. Materials and Methods

### 2.1. Ethics and Recruitment Model

This study was approved by the Ethics Committee of the Medical University of Bialystok, Poland (R-I-002/307/2019), registered at Clinical Trials.gov (NCT 04227223), and performed in accordance with the Declaration of Helsinki. Adult patients who met low back pain criteria and received opioid therapy as a polytherapy scheme were included. Patients with cognitive disorders, renal insufficiency, metabolic diseases, cancer pain, and antioxidant supplementation were excluded. The control group included patients with LBP who did not receive any pharmacotherapy.

The procedure was fully explained to each participant, and all patients signed an informed consent form. Then, the participants were surveyed for demographic information such as age, gender, and anthropometric parameters, including weight and height. The participants identified their most significant pain intensity over the past week, and their actual pain severity (at the current moment) was assessed using the numerical rating score (NRS) score in the range of 0–10. Pain intensity was assessed using the WHO classification of mild (NRS 1–3), moderate (4–6), and severe pain (7–10), based on pain intensity over the past week. Medical information about pharmacotherapy was recorded. Typical multimodal opioid-based analgesia means opioids combined with typical analgesics from Steps 1 and 2 of the WHO Analgesic Ladder. Adjuvant multimodal opioid-based analgesia means opioids combined with non-specific analgesics. The morphine milligrams equivalents (MMEs) were calculated based on doses of opioids. The Douleur Neuropathique 4 questionnaire (DN4), which includes 10 pain-related items based on interviews and clinical examinations, was used to diagnose neuropathic pain. Scores of four or more were considered to indicate neuropathic pain, while scores less than four were considered non-neuropathic pain.

**Figure 1:**
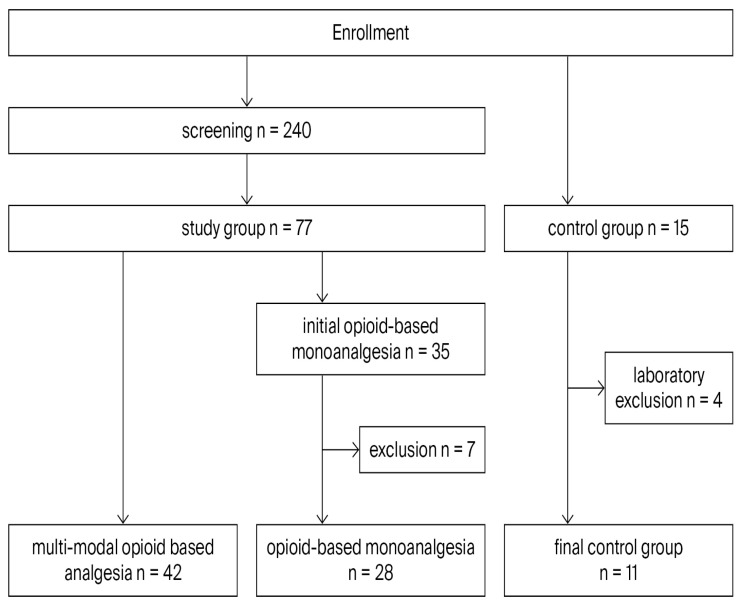
A flow chart of the recruitment model.

In this study, a multi-step recruitment process was implemented. In the first stage, we screened the medical records in Pain Clinic, who met the criteria for LBP, and then we selected records with multimodal opioid-based analgesia therapy. In the next stage, we included patients with a regular prescription for opioid medications, and we considered this criterion as fundamental. Additionally, we excluded records with multimodal analgesia regimens consisting of both typical and adjuvant analgesics. A flow chart of the recruitment model is presented below (Figure 1).

### 2.2. Characteristics of Patients

The polytherapy group consisted of 20 female and 22 male patients, with a median age of 72 years and a BMI of 24.8. This group consisted of 18 patients on a typical analgesia scheme and 24 patients on an adjuvant analgesia scheme. The most popular typical analgesia scheme consisted of opioid/paracetamol/diclofenac and was noted in 35% patients. Additionally, the opioid/pregabalin/steroids combination was noted in 45% of the patients on an adjuvant analgesia scheme. The median morphine milligram equivalents in the multimodal opioid-based analgesia model was 42, with a min–max range of 15–135.

The multimodal opioid-based analgesia used oxycodone in 46% of cases, buprenorphine in 26%, fentanyl in 8%, and tramadol in 12%. Oral opioids were used more frequently (84%) than transdermal patches. Non-opioid analgesics were used in 79% of cases, including paracetamol in 53%, metamizole in 22%, ibuprofen in 15%, ketoprofen in 14%, and diclofenac in 35%. The most commonly used co-analgesics were pregabalin and steroids. Muscle relaxants were used in 25% of multimodal opioid-based analgesia cases.

Oxycodone was the most frequently used opioid in typical and adjuvant multimodal polytherapy. The median duration of analgesic polytherapy was 22 months, with a min–max range of 8–26 months. The median equivalent of morphine was 36 mg (IQR: 15–96 mg of morphine) for typical polytherapy analgesia and 34 mg (IQR 12–86 mg) for the adjuvant model of opioid-based polytherapy analgesia. A mild-pain severity score (NRS 1–3) was reported in 19 patients, a moderate-pain severity score (NRS 4–6) in 14 patients, and a severe-pain score (NRS 7–10) in 9 patients in the polytherapy group.

The data from the polytherapy group did not differ in age and BMI parameters. The females made up the dominant part of the control group at 54%, 47% of the polytherapy group and 35% of the monotherapy group (Table 1).

### 2.3. Laboratory Assessment

Following clinical assessment, the researchers collected blood samples via venipuncture (21-gauge needle, BD vacutainer, Franklin Lakes, NJ, USA). The blood samples were aseptically drawn into ethylenediaminetetraacetic acid tubes (2.7 mL EDTA BD vacutainers, Franklin Lakes, NJ, USA). Within 30 min of collection, the samples were centrifuged (1000× *g* for 15 min at 2–8 °C) and immediately stored at −80 °C in 300-microliter aliquots in Eppendorf tubes until required. BDNF analysis was performed using a high-sensitivity, human-specific BDNF enzyme-linked immunosorbent assay (ELISA, Immunodiagnostik, Bensheim, Germany). Plasma BDNF values were reported in ng/mL. The coefficients of variance ranged between 2.9% and 8.1%, the lower limit of quantification was 0.61 pg/mL, and the intra- and inter-assay coefficients of variation were <10%. The potential effect of variation between plates/kits was eliminated by ensuring that all samples from each participant were measured using the same plate/kit. The room temperature during the analyses ranged from 23.7 to 26.2 °C. Sample processing and data analysis were performed according to the manufacturer’s instructions (Immundiagnostik, Bensheim, Germany).

### 2.4. Statistical Analysis

The Shapiro–Wilk test was used to assess the normality of the variable distributions. In further analyses, non-parametric tests were used to identify correlations between variables and between groups. The data are presented as the median, minimum–maximum range, and 25–75th percentile range (IQR), or as counts (n) with proportions (%).

The Kruskal–Wallis test was used to compare BDNF values between three independent groups, while the Mann–Whitney U test was used to compare two independent groups. The correlation between BDNF and pain intensity in neuropathic pain was analyzed using Spearman’s rank test. An ROC analysis was performed to determine and compare the sensitivity and specificity of BDNF as a predictor of pain intensity and neuropathic pain. Multiple linear regression was used to analyze the mechanisms and correlations between BDNF and factors related to sex, adjuvant and typical analgesia, and neuropathic pain. All calculations were performed using Statistica 14.0.0 (TIBCO Software Inc., Cracow, Poland), and *p* < 0.05 was used as the level of significance.

## 3. Results

The analysis presented that the polytherapy group had significantly higher BDNF values than the monotherapy group (*p* = 0.01). Additionally, no statistical differences were observed compared to the control group (*p* = 0.75). The median plasma BDNF value in males was 3.9 ng/mL (IQR 2.9–6.4, min–max 1.8–8.2) and did not differ significantly compared to females—3.1 ng/mL (IQR 2.78–4.74, min–max 2.02–8.96), *p* = 0.51.

In the study, no significant differences in BDNF values were observed between the polytherapy group using the typical analgesic model and the adjuvant analgesic model: 3.7 ng/mL (IQR 2.7–6.9, min–max 2.4–8.2) vs. 3.6 ng/mL (IQR 2.8–4.7, min–max 1.8–8.9), *p* = 0.53, respectively.

The study found that median BDNF values were lowest in the mild-pain group and increased with increasing pain intensity, as measured by the NRS, with the severe-pain group showing the highest median BDNF values. The median, minimum, and maximum ranges, as well as the IQRs, are presented in Table 2.

**Figure 2:**
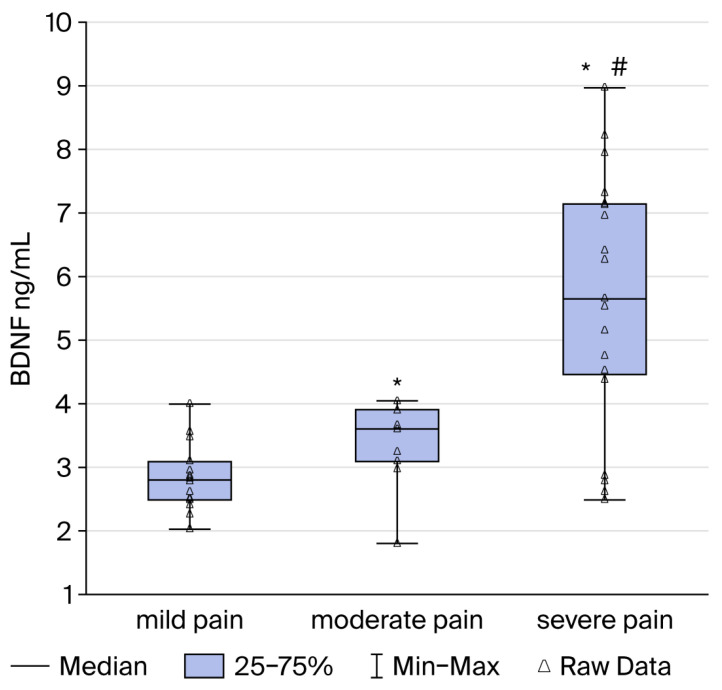
The BDNF in mild-, moderate- and severe-pain scores by NRS. * compared to mild pain, *p* < 0.05. # compared to moderate pain, *p* < 0.05.

Significant differences in BDNF values were observed between the severe-pain and moderate-pain groups (*p* = 0.006) and the mild-pain group (*p* = 0.0001) (Figure 2).

**Figure 3:**
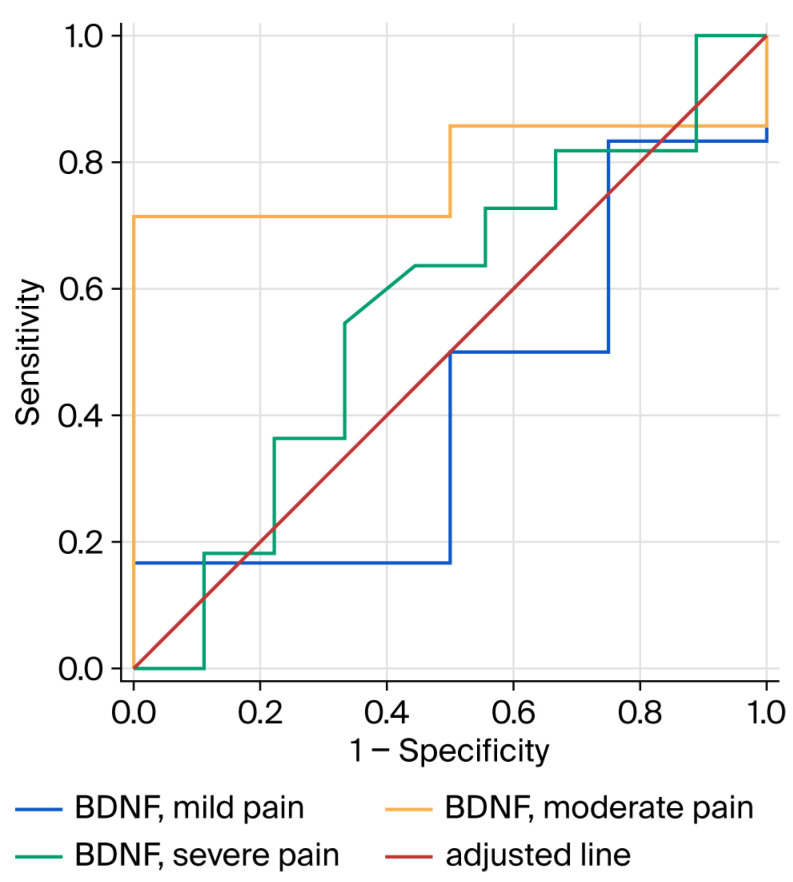
ROC curve for BDNF in mild, moderate and severe pain.

The ROC analysis indicated that plasma BDNF values did not predict significant pain intensity in multimodal opioid-based analgesia. Figure 3 presents the ROC curves for BDNF in mild, moderate, and severe pain. The cut-off points, AUC values, and *p*-values are presented in Table 3.

**Figure 4:**
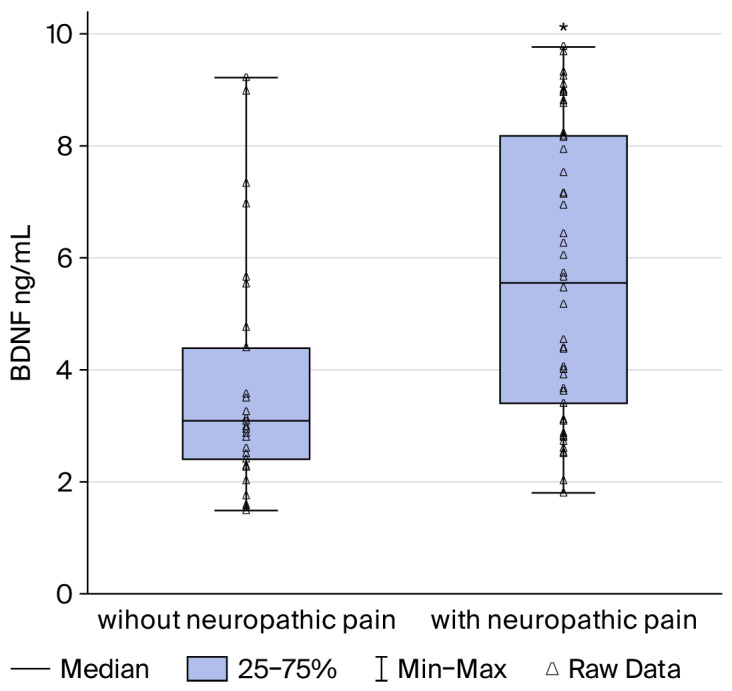
BDNF in neuropathic pain. * significant with *p* < 0.05.

The analysis showed that in the patient group with neuropathic pain, the median BDNF value was significantly higher than that in the group without neuropathic pain (*p* = 0.0005). The median, minimum, and maximum ranges, as well as the IQRs, are presented in Table 2. Figure 4 presents the statistical significance of BDNF in the neuropathic-pain group.

**Figure 5:**
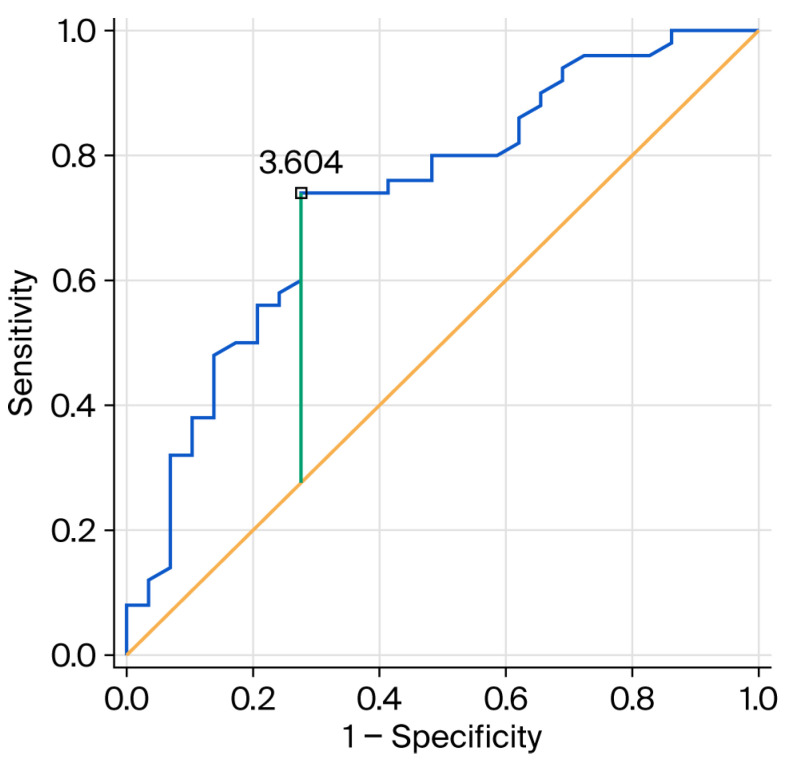
The ROC curve for BDNF in neuropathic pain in multimodal opioid-based analgesia.

A strong, statistically significant correlation was found between BDNF and NRS in the neuropathic-pain component (rho = 0.6, *p* = 0.001). The ROC analysis indicated that a plasma BDNF value greater than 3.604 ng/mL significantly predicted neuropathic pain in patients receiving multimodal opioid-based analgesia (AUC 0.73, 95% AUC 0.62–0.85, Youden Index 0.46, *p* = 0.0001, sensitivity 74%, and specificity 72%). The ROC curve for BDNF in predicting neuropathic pain in patients receiving multimodal opioid-based analgesia is presented in Figure 5.

Multiple linear regression identified a significant increase in the NRS depending on plasma BDNF in the typical and adjuvant analgesia models, in the coexisting neuropathic-pain component, and in the male and female groups. The analysis details are presented in Table 4.

## 4. Discussion

Our study uncovered crucial results. First, BDNF values were significantly lower in patients receiving multimodal opioid-based therapy analgesia than in those receiving opioid monotherapy, but not significantly different from the controls. Second, BDNF values were significantly higher in patients with moderate and severe pain than in patients with mild pain. Moreover, the median BDNF value was significantly higher in patients with neuropathic pain within the multimodal opioid-based analgesia model. Additionally, BDNF values were correlated with neuropathic pain intensity. A plasma BDNF value greater than 3.604 ng/mL significantly predicted neuropathic pain in patients receiving multimodal opioid-based analgesia (AUC 0.73, *p* = 0.0001, sensitivity 74%, and specificity 72%).

The relationship between chronic pain and circulating BDNF levels has scarcely been investigated, with studies mainly focusing on fibromyalgia, migraine, osteoarthritis, and musculoskeletal pain. However, many of these studies are limited by small sample sizes and incomplete factor analyses (therapy, lifestyle factors, anthropometrical data, sex-related factors, physical activity, etc.) [23,24,25,26].

To the best of our knowledge, this is the first study to compare BDNF between different pharmacological models of analgesia in chronic low back pain.

The mean BDNF concentration in patients with osteoarthritis was 296 pg/mL, and further analysis showed that plasma BDNF concentration was significantly correlated with pain intensity on the NRS scale [27]. Simão et al. reported that BDNF levels in the synovial fluid of patients with osteoarthritis were sixfold lower than in that of controls and fourfold lower than in control plasma [28,29].

Kamel et al. demonstrated that serum BDNF levels in patients with discogenic chronic LBP were significantly higher than in a control group. ROC analysis also indicated that serum BDNF levels above 1201 ng/mL, with a PPV of 79%, indicated discogenic pain (AUC 0.83, *p* < 0.05, sensitivity 77, specificity 80, NPV 78). Serum BDNF levels were also significantly higher in a neuropathic-pain group with concomitant chronic pain (1.26 ng/mL vs. 1.20 ng/mL, *p* = 0.03). The authors found no relationship between NRS pain intensity and BDNF (*p* = 0.42) but did indicate such a relationship with acute pain intensity (*p* = 0.026) [30].

This is also consistent with the observations of Sobański et al., who demonstrated that BDNF concentrations in spinal disk tissue during chronic pain were significantly higher, reaching 28 pg/mg compared to 4.5 pg/mg in a control group (*p* < 0.05) [31].

Other studies conducted in patients with rheumatoid arthritis demonstrated that serum BDNF concentrations were significantly lower in the active and inactive forms of the disease compared to a control group, reaching 1.58 ng/mL vs. 1.17 ng/mL vs. 3.01 ng/mL, respectively (*p* < 0.001). ROC analysis showed that BDNF values < 2 ng/mL were characteristic of patients with active rheumatoid arthritis, with a sensitivity of 80% and a specificity of 96.7% [32].

In addition, among older women after an acute episode of LBP, plasma BDNF concentrations were significantly higher than in controls (7515 vs. 6331 pg/mL, *p* = 0.005). The median BDNF values were 7455 in patients with mild pain (*p* = 0.005), 6597 in patients with moderate pain (*p* = 0.044), and 7410 in patients with severe pain (*p* = 0.03), all of which were statistically higher than the control value (5897). However, no statistical differences were observed between subgroups. Similar relationships were found for pain intensity using past-week data. The median values were 7647 in patients with mild pain (*p* = 0.024), 7275 in patients with moderate pain (*p* = 0.022), and 6833 in patients with severe pain (*p* = 0.029), compared to a control value of 5897 [33]. Similarly, Simao et al. reported a positive correlation between plasma BDNF and pain intensity in elderly people with acute osteoarthritis pain (rho = 0.39, *p* = 0.04) [28,29].

Another analysis showed that in both the opioid and non-opioid analgesia groups, median plasma BDNF values were significantly higher than those in the control group, reaching 7013 (*p* = 0.008 vs. 7195 and *p* = 0.19 vs. 5897, respectively). However, no differences were observed between the subgroups for plasma BDNF concentration (*p* = 0.589) [33]. Gender, anthropometric factors (BMI), comorbid psychiatric conditions (depression), metabolic conditions, and laboratory test methods are important factors that influence BDNF concentrations, although the published data are variable [31,34,35,36,37]. Diaz et al. found no correlation between plasma BDNF and age, BMI, physical activity, comorbidities, or medications taken in an LBP group [33].

Ortola et al. also found no significant differences in BDNF values between a study group with comorbid depression and a healthy control group: 19.7 vs. 18.8 ng/mL. However, among men with chronic pain and depression, the median values were lower, and a significant correlation was found between the reduction in BDNF values and the severity of depression, with a decrease of 0.6 ng/mL per one point of the GDS depression score. No such correlation was found for women with chronic pain (0.12 ng/mL per 1 GDS point). Among women with chronic pain without depression, a significant correlation was found, with a 0.45 ng/mL decrease in BDNF for each one-point increase in the VAS. Among men with chronic pain without depression, the correlation was a 1.53 ng/mL decrease in BDNF for each one-point increase in the VAS pain score [38].

BDNF concentrations in chronic pain also depend on psychological factors and physical activity. It has been shown that the use of physical activity programs in chronic pain resulted in a significant increase in BDNF (2.174 vs. 3.063, *p* = 0.001); however, this was not associated with a reduction in pain intensity [39,40,41,42]. Another study showed that educational activities did not significantly increase BDNF but significantly reduced the intensity of chronic pain. For each 1 pg/mL increase in the Δ BDNF, there was a 0.32 kg increase in Δ IQMS (standardized regression coefficient = 0.57; R squared = 0.32; *p* = 0.03) [28,29].

In our study, plasma BDNF values did not differ significantly between males and females. Increasing plasma BDNF in females resulted in lower pain intensity than in males (b = 0.79 vs. b = 1.03, respectively). Multivariate linear regression analysis indicated that adjuvant analgesia had a more favorable effect on the relationship between pain intensity and BDNF levels. A 1 ng/mL increase in BDNF resulted in a 0.63 increase in NRS compared to the typical analgesia model (the NRS increased by 1.38 per 1 ng/mL of BDNF). In both neuropathic and non-neuropathic pain, the NRS increase due to BDNF was similar, reaching 0.74 and 0.89, respectively.

Laboratory analyses show that BDNF freely diffuses across the blood–brain barrier and reaches comparable concentrations in blood; therefore, blood tests are used as a marker of BDNF neuronal status. Blood BDNF is detectable in plasma and serum. Although plasma BDNF is quite unstable, sensitive to preparation procedures, and reflects the body’s physiopathological conditions, it correlates positively with platelet activation state. Conversely, serum BDNF is more stable in laboratory measurements but is modified by temperature and coagulation. The total serum BDNF value represents the total amount of BDNF released from platelet activation after blood clotting [36,43,44,45]. In our study we used plasma measurements because of reduction risk of coagulation mistakes.

Hajer et al. found that salivary BDNF also varied rhythmically, with statistically higher levels in the morning. Moreover, patients with myalgia had significantly lower salivary BDNF compared to controls, and BDNF values were correlated with psychological dysfunction [46]. In another study, the authors demonstrated significant differences in BDNF between various fractions of stimulated and unstimulated saliva but no significant correlations between total BDNF levels in saliva and plasma [47]. Indeed, concerning methodological aspects, it is well known that anticoagulation, temperature, delayed centrifugation, and long-term storage influence BDNF measurements. Additionally, ELISA kits have different detection ranges, sensitivities, and inter-assay variations [46,47,48,49,50].

Based on recommendations, opioids are a possible treatment for severe acute postoperative pain, but multimodal postoperative analgesia with limited opioid use is preferred [51,52,53]. Moreover, opioid therapy is not considered to be the first-line treatment for chronic pain [54,55]. Additionally, many national and international institutions and societies recommend limiting the use of opioids [56,57,58,59]. The Centers for Disease Control and Prevention (CDC), part of the US Department of Health and Human Services, presented 12 recommendations in 2016. Firstly, non-opioid therapy is preferred for the treatment of chronic pain, and opioids should be used only when their benefits for pain and function are expected to outweigh the risks. Secondly, opioid therapy needs to be planned, establishing treatment goals with patients and preparing them for how the opioids will be discontinued if their benefits do not outweigh the risks. Moreover, the opioids should be prescribed at the lowest effective dosage, ideally below 50 morphine milligram equivalents per day, and concurrent use of opioids and benzodiazepines should be avoided, with evaluation of the effects every 3 months (or more frequently), and prescriptions should be reviewed for high-risk combinations or dosages [54]. In 2022, the CDC presented the following categories of recommendations: determining whether or not to initiate opioids for pain, selecting the opioids and determining their dosages, deciding the duration of the initial opioid prescription and conducting follow-up, assessing risk, and addressing potential harms of opioid use [55]. The American Society of Interventional Pain Physicians presented comprehensive, evidence-based consensus guidelines for the prescription of opioids for chronic non-cancer pain and finalized 20 guidelines for opioid administration, including four sections specific to opioid therapy, with 10 recommendations particular to the initial steps of opioid therapy, 5 recommendations for assessing the effectiveness of opioid therapy, 3 recommendations about monitoring adherence and side effects, and 2 general recommendations [60]. It is crucial to maximize the use of non-pharmacological and non-opioid pharmacological therapies, as appropriate for the specific condition and patient, and to only consider initiating opioid therapy if the expected benefits for pain and function are anticipated to outweigh the risk to the patient [54,55,60].

Our study utilized an existing medical database for analysis. Furthermore, the recruitment process did not assume the implementation of opioid medications. An assessment of available treatment methods for LBP episodes revealed that the first stage of medical intervention is non-invasive therapeutic methods, which were used by a total of 49% of the participants (chiropractic therapy, cognitive therapy, exercise therapy, and physical therapy), on average 39 days after diagnosis. The next step in medical intervention is the initiation of pharmacotherapy, which was observed in 69% of the participants approximately 50 days after diagnosis. The earliest use was observed for skeletal muscle relaxants (27% of the participants, 52 days after diagnosis), NSAIDs (35% of the participants, 73 days after diagnosis), and antidepressants (22% of the participants, 79 days after diagnosis). The use of opioid drugs was more frequent than the presented pharmacological groups and accounted for 41% of cases, including strong opioids (39%) and weak opioids (6.6%), implemented on average 82 days after diagnosis [61].

In chronic LBP, the use of non-invasive therapeutic methods remained comparable, accounting for approximately 46% of cases. However, a significant increase in the frequency of pharmacotherapy use was noted, reaching 84%. The analysis revealed that 73% of the study participants were prescribed opioids, including 70% for strong opioids and 15% for weak opioids. The frequency of NSAID use increased slightly, accounting for 43% of cases. A similar trend was observed in the use of systemic corticosteroids and skeletal muscle relaxants. In chronic LBP, the use of anti-epileptic drugs increased compared to acute LBP episodes (17.3 vs. 8.4%), as did the use of antidepressants and benzodiazepines (24 vs. 22, and 18 vs. 15%, respectively) [61]. Epidemiological data based on the Healthcare for Communities (2000–2001) survey showed that the decision to use opioid medications was associated with the occurrence of pain limiting daily functioning (57%) and poor quality of life (25%). Arthritis, back pain, and chronic headaches were the most prevalent conditions for opioid-based pharmacotherapy (63% vs. 59% vs. 38%, respectively). Further analyses indicated that patients aged 18–29 years, those with complex pain and comorbid mental health disorders, and those with moderate-to-severe pain interference used opioids more frequently [62].

In the study by Deyo et al., 24% of patients reported episodic preoperative opioid use, and 5% reported short-term opioid use. Long-term opioid use predominated (43%), and 77% continued postoperatively. A small group (13%) reported episodic postoperative opioid use, and 9% discontinued opioid therapy. In the group of patients receiving postoperative opioids, 32% used high doses—defined as more than 90 morphine milligram equivalents (MMEs)/day—while 11% maintained a stable dosage, and 34% reduced to lower doses (less than 90 MMEs). Regression analysis showed that preoperative opioid use and a cumulative preoperative dose greater than 8100 MMEs in the previous 7 months or a mean daily dose greater than 39 MMEs (OR 15.47) are significant risk factors for continuing therapy postoperatively [63]. Connoly et al. indicated that additional risk factors include the use of opioids within the year before surgery (OR 2.27), refusion surgery (OR 1.32), and a diagnosis of depression (OR 1.43) [64]. Opioid therapy for the management of LBP is most often initiated by hospital referral, the emergency department, or primary care [54,55,65,66,67].

The goal of a multimodal pain management strategy is to delay the use of opioid medications and to reduce their dosage and side effects. The fundamental principle is based on combining the synergistic pharmacological effects of non-opioid analgesics and co-analgesics with multidirectional mechanisms of action. The literature has highlighted the significant impact of antidepressants and antipsychotics on BDNF levels, while typical analgesic agents have less important activity. Referring to previous analyses from preliminary studies, we demonstrated that BDNF levels were higher in the multimodal opioid-based analgesia group compared to opioid monotherapy. Further analysis showed that BDNF levels did not differ significantly between typical and adjuvant analgesia. Thus, it can be concluded that opioid medications are the cause of the changes in BDNF.

The present study has some limitations that need to be considered when interpreting the results. First, because this study was a secondary analysis, we were restricted to data obtained as a part of the original study. The size of the study groups depended on database availability. Due to the numerous and understandable limitations of opioid therapy, we used the medical database available at the Pain Clinic. The study protocol did not assume recruitment of new patients.

The methodology of the study involved a multi-stage screening of available medical records, and the study group was selected on this basis. To limit the heterogeneity of the study group, we excluded records involving a combination of typical and adjuvant multimodal analgesia during the recruitment process. Of course, such pharmacological combinations also have clinical relevance and may be interesting for the continuation of the project. We applied a phased screening of available records to construct a model for the evaluation of opioid medications. The first objective was to compare BDNF levels between polytherapy and monotherapy, and the second objective was to determine whether there are differences in BDNF levels in analgesia models using typical and adjuvant co-analgesics.

However, our results should be interpreted cautiously due to the small sample size. While the results of this study lay a solid basis for subsequent research, future studies should consider these limitations by enrolling larger samples to enhance sensitivity. Second, the polytherapy regimens were so diverse that it was difficult to analyze individual models, making it impossible to assess the synergistic effects of various adjuvant and typical analgesics. Furthermore, the study group was characterized by high variability in clinical characteristics and a variety of analgesic regimens combining opioids with co-analgesics and adjuvant medications. Our study was exploratory and clinical aspects need external validation in independent studies.

In our study, the potential effect of variation between plates/kits was eliminated by ensuring that all samples from each participant were measured using the same plate/kit. The room temperature during the analyses ranged from 23.7 to 26.2 °C. The recruitment and blood sampling took place in the morning. Despite efforts to control external variables, such as circadian rhythms, blood sample collection, and temperature, these measurements may not have been sufficient to eliminate all factors.

## 5. Conclusions

The neurotrophin BDNF is a crucial factor involved in nociception and pathological mechanisms of chronic and neuropathic pain. Little is known about circulating BDNF and analgesia models. Opioids are the strongest multidirectional analgesics, with specific neuroapoptosis and neurodegeneration side effects. To the best of our knowledge, this study presents the first original analysis investigating the association between circulating plasma BDNF levels and pain pharmacotherapy in chronic non-cancer pain.

However, our study was exploratory and results should be interpreted cautiously. Clinical aspects need external validation in independent studies.

Multimodal opioid-based analgesia decreases plasma BDNF concentrations less than opioid monotherapy, which offers an opportunity to limit the adverse effects of opioid-induced neuroapoptosis.

BDNF concentration influences pain intensity and the neuropathic-pain component. Despite the use of an advanced analgesic model, plasma BDNF concentrations remained consistently high in patients with severe pain and neuropathic pain, confirming BDNF’s significant role in the pathogenesis of chronic pain. BDNF predicted the occurrence of a neuropathic-pain component in chronic pain.

## Figures and Tables

**Table 1:** Characteristics of patients included in the study. * statistical significance with *p* < 0.05, monotherapy compared to controls. # statistical significance with *p* < 0.05, polytherapy compared to monotherapy.

Parameter	Polytherapy	Monotherapy	Control Group
**Age** **[median, min–max range]**	72.6 (47–76)	70.5 (43–80)	64.0 (30–81)
**Sex** **[n, female/male]**	42 (20/22)	28 (10/18)	11 (6/5)
**BMI** **[median, min–max range]**	24.8 (19.7–31.3)	27.2 (21.2–36.3)	26.7 (21.3–34.6)
**BDNF ng/mL** **[median, min–max range, IQR]**	3.6 1.8–8.9 2.7–5.6 #	2.7 1.1–4.8 2.2–3.1 *	5.0 3.9–9.0 4.5–7.5

**Table 2:** The BDNF in mild, moderate and severe pain in polytherapy. The median, min–max ranges and IQR are presented.

	BDNF ng/mL		
	Median	Min–Max Range	IQR
**Mild pain**	2.8	2.1–3.9	2.4–3.1
**Moderate pain**	3.60	1.8–4.0	3.1–3.9
**Severe pain**	5.64	2.4–8.9	4.4–7.1
**With neuropathic pain**	5.5	1.8–9.7	3.4–8.1
**Without neuropathic pain**	3.1	1.49–9.2	2.4–4.3

**Table 3:** The cut-off points, AUC values, 95% AUC, Youden Index and p based on ROC analysis.

Pain Intensity	BDNF Cut-Off Value	AUC	CI 95% AUC	Youden Index	*p*
**Mild**	2.03 (ng/mL)	0.41	0.09–0.74	0.17	0.61
**Moderate**	2.61 (ng/mL)	0.78	0.48–1	0.71	0.06
**Severe**	3.6 (ng/mL)	0.57	0.3–0.83	0.21	0.59

**Table 4:** The details of multiple linear regression analysis of BDNF (independent factor) and NRS (dependent factor).

Factor	b	t	*p*
**Adjuvant analgesia**	0.63	2.86	0.008
**Typical analgesia**	1.38	11.7	0.0001
**Without neuropathic pain**	0.89	3.59	0.02
**With neuropathic pain**	0.74	3.55	0.017
**Male**	1.03	5.20	0.0005
**Female**	0.79	3.08	0.005

## Data Availability

Data are unavailable due to privacy or ethical restrictions.
